# Glycine decarboxylase deficiency causes neural tube defects and features of non-ketotic hyperglycinemia in mice

**DOI:** 10.1038/ncomms7388

**Published:** 2015-03-04

**Authors:** Yun Jin Pai, Kit-Yi Leung, Dawn Savery, Tim Hutchin, Helen Prunty, Simon Heales, Margaret E. Brosnan, John T. Brosnan, Andrew J. Copp, Nicholas D.E. Greene

**Affiliations:** 1 Birth Defects Research Centre and Developmental Biology & Cancer Programme, Institute of Child Health, University College London, London WC1N 1EH, UK; 2 Newborn Screening and Biochemical Genetics, Birmingham Children’s Hospital, Birmingham B4 6NH, UK; 3 Department of Chemical Pathology, Institute of Child Health, University College London, Great Ormond Street Hospital for Children NHS Foundation Trust, London WC1N 3JH, UK; 4 Department of Biochemistry, Memorial University of Newfoundland, St John’s, Newfoundland and Labrador, Canada A1B3X9

## Abstract

Glycine decarboxylase (GLDC) acts in the glycine cleavage system to decarboxylate glycine and transfer a one-carbon unit into folate one-carbon metabolism. *GLDC* mutations cause a rare recessive disease non-ketotic hyperglycinemia (NKH). Mutations have also been identified in patients with neural tube defects (NTDs); however, the relationship between NKH and NTDs is unclear. We show that reduced expression of *Gldc* in mice suppresses glycine cleavage system activity and causes two distinct disease phenotypes. Mutant embryos develop partially penetrant NTDs while surviving mice exhibit post-natal features of NKH including glycine accumulation, early lethality and hydrocephalus. In addition to elevated glycine, *Gldc* disruption also results in abnormal tissue folate profiles, with depletion of one-carbon-carrying folates, as well as growth retardation and reduced cellular proliferation. Formate treatment normalizes the folate profile, restores embryonic growth and prevents NTDs, suggesting that Gldc deficiency causes NTDs through limiting supply of one-carbon units from mitochondrial folate metabolism.

## Abstract (web summary)

Mutations in the enzyme glycine decarboxylase (GLDC) are associated with neural tube closure defects and non-ketotic hyperglycinemia in humans. Here the authors generate a mouse model with reduced Gldc expression and activity and study the direct effect of the enzyme in these diseases and the mechanisms responsible for neural tube closure defects.

## Graphical abstract

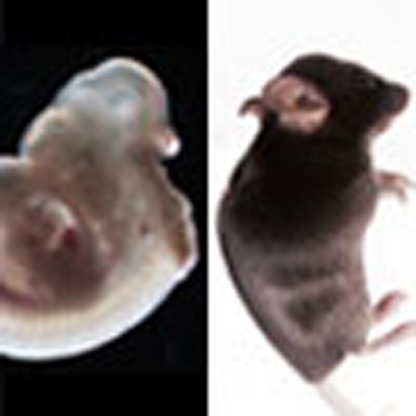

The glycine cleavage system (GCS) is a multienzyme complex that mediates the breakdown of glycine in mitochondria (Fig. 1a). Glycine decarboxylase (GLDC; glycine dehydrogenase (decarboxylating)), encoded by *GLDC*, catalyses the first step of glycine cleavage, in which one carbon is released as CO_2_. This reaction occurs in the presence of an accessory protein, GCS H-protein (*GCSH*), to which the aminomethyl moiety is transferred^1^. The subsequent action of aminomethyltransferase (*AMT)* transfers the second one-carbon unit to tetrahydrofolate (THF), generating 5,10-methylene THF. The further steps of mitochondrial folate one-carbon metabolism (FOCM) ultimately supply one-carbon units, as formate, to the cytoplasm for several functions including nucleotide biosynthesis and methylation reactions^2^. Isotope-tracing analysis of glycine flux in adult humans suggests a major contribution of glycine-derived one-carbon units to FOCM and serine synthesis^3^.

Abnormal overexpression of *Gldc* has been associated with lung cancer and other tumours^4^. In comparison, loss-of-function mutations in GCS-encoding genes cause the rare autosomal recessive disease, non-ketotic hyperglycinemia (NKH), also known as glycine encephalopathy (OMIM no. 605899), characterized by elevated glycine in body fluids. NKH occurs with a frequency of ~1/60,000 in Canada and 1/12,000 in Finland, with higher rates in some populations of Israel and the Netherlands^5^,^6^. Signs of NKH typically present in neonates, with progressive muscular hypotonia, myoclonic jerks, respiratory problems (apnea) and coma. Around one-third of patients die within the first year^5^,^7^. Patients who survive the neonatal period (with assisted breathing) or present later, as infants, have profound neurological impairments with intractable seizures, developmental delay and psychomotor retardation^5^,^8^. Other features found in some patients include acquired hydrocephalus and/or absence of the corpus callosum^7^. A mouse model for NKH has not been reported to date.

In addition to causing NKH, mutations in GCS-encoding genes have been proposed to predispose to neural tube defects (NTDs), common birth defects of the developing central nervous system (CNS), which include spina bifida and anencephaly^9^,^10^. NTDs occur in around 1 per 1,000 pregnancies worldwide and have a complex multifactorial causation, involving multiple genetic and environmental factors. GCS genes represented candidates for involvement in NTDs on the basis of evidence implicating FOCM as a determinant of susceptibility to NTDs. For example, maternal use of folic acid supplements during early pregnancy has a significant protective effect^11^. Conversely, suboptimal maternal folate status is a risk factor for NTDs, and abnormalities of FOCM have been identified in some patients^12^,^13^. We identified missense mutations in *GLDC* and *AMT* in NTD patients, and NTD-specific GLDC variants were found to compromise enzyme activity^14^. Mice lacking Amt function were found to develop NTDs^14^; however, the metabolic and cellular mechanism underlying these defects are yet to be determined. Mice lacking Gldc function have not been reported; therefore, it is not yet proven that this protein is essential for neural tube closure.

An association of NKH and NTDs has not been reported in humans. Mutations reported in NTD patients were present in heterozygous form, which would be insufficient to cause NKH^14^, while cranial NTDs are lethal at birth, so co-existing NKH would not be recognized. The almost complete penetrance of NTDs in *Amt* mutant mice precluded the investigation of possible NKH-like phenotypes.

In the current study, we generated a novel mouse model with reduced *Gldc* expression and lack of glycine cleavage activity. Homozygous mutants display NTDs and NKH-like phenotypes together with metabolic derangement of glycine and FOCM. These findings suggest that both glycine decarboxylation and transfer of a one-carbon group into FOCM are essential for normal embryonic development and post-natal brain function.

## Results

### Generation of mice lacking Gldc function

**Figure 1:**
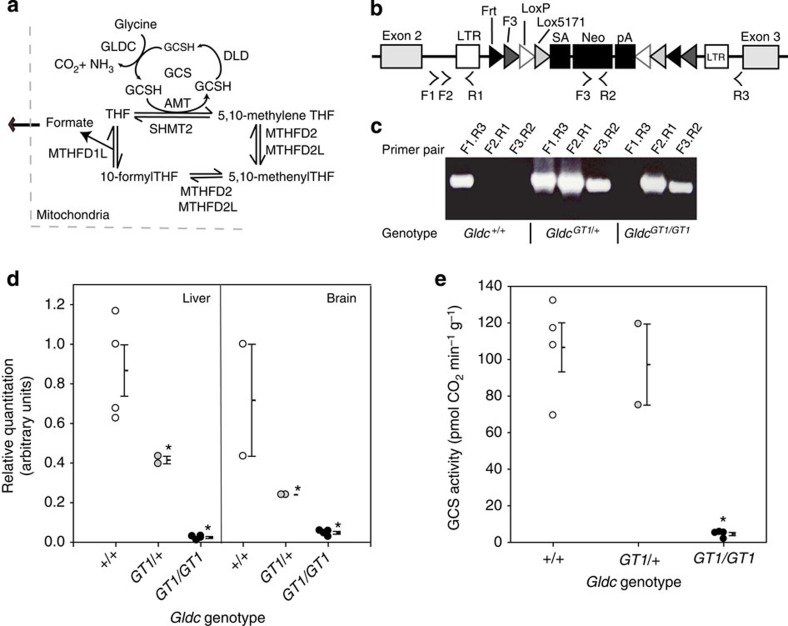
Gene trap of *Gldc* ablates GCS activity. The GCS, comprising GLDC, GCSH, AMT and DLD (dihydrolipoamide dehydrogenase), functions in mitochondrial folate metabolism (**a**) to cleave glycine, donating a one-carbon unit to THF. The *Gldc*^*GT1*^ allele (**b**) carries a gene-trap construct in intron 2. Splicing of exon2 to the gene-trap splice acceptor, SA, generates a truncated mRNA. The primers used for genotyping by PCR of genomic DNA are indicated (**b**). F1 and R3 generate a band in wild-type and heterozygous mice that is not detectable in homozygous *Gldc*^*GT1/GT1*^ mice (**c**). No PCR product is generated in +/+ samples when one or both primers are located within the trap construct (F2.R1 or F3.R2; full image of gel is included in Supplementary Information). (**d**) Quantitative real time RT–PCR analysis of liver and brain samples at 5 weeks of age. (**e**) Enzymatic activity of the GCS in liver (* indicates significant difference from *Gldc*^*+/+*^, *P*<0.001 analysis of variance (ANOVA); error bars represent s.e.m. throughout the manuscript).

We generated mice carrying a gene-trap allele of *Gldc*, using an embryonic stem cell line carrying a trap construct in intron 2 (*Gldc*^*GT1*^; Fig. 1b). The insertion site and structure of the gene-trap cassette was verified by sequencing of genomic DNA and mice were subsequently genotyped by PCR of genomic DNA (Fig. 1c). Alternative splicing to the splice acceptor in the construct was confirmed by reverse transcriptase–PCR (RT–PCR) and generates a truncated transcript lacking exons 3–25, predicted to be functionally null. Heterozygous *Gldc*^*GT1/+*^ mice were viable and fertile, and we therefore intercrossed heterozygotes to generate homozygous *Gldc*^*GT1/GT1*^ offspring. Initial analysis of pups, genotyped at 10 days of age, showed that *Gldc*^*GT1/GT1*^ mice were viable. Among 15 litters of mice, a smaller number of homozygous pups were present than would be predicted by the number of heterozygotes (*n*=20 *Gldc*^*+/+*^, 49 *Gldc*^*GT1/+*^, 15 *Gldc*^*GT1/GT1*^). Although this difference did not represent a statistically significant variation from the Mendelian ratio (*P*>0.05, *χ*^2^-test) the presence of ~35% fewer homozygotes than expected raised the possibility of fetal or perinatal lethality, which was subsequently confirmed (see below).

We confirmed that gene-trapping of *Gldc* was effective in post-natal mice by analysis of mRNA from adult brain and liver at 5 weeks of age. This showed a 50–60% reduction in expression in heterozygotes and 95% reduction of expression in homozygous mutants (Fig. 1d). In accordance with loss of *Gldc* expression, measurement of the enzymatic activity of the GCS, by assay of glycine decarboxylation, showed that activity was almost undetectable in homozygous mutant mice compared with wild-type littermates, whereas 50% mRNA production was sufficient to produce normal liver enzyme activity in heterozygotes (Fig. 1e).

### Gldc deficiency causes elevated glycine levels

**Figure 2:**
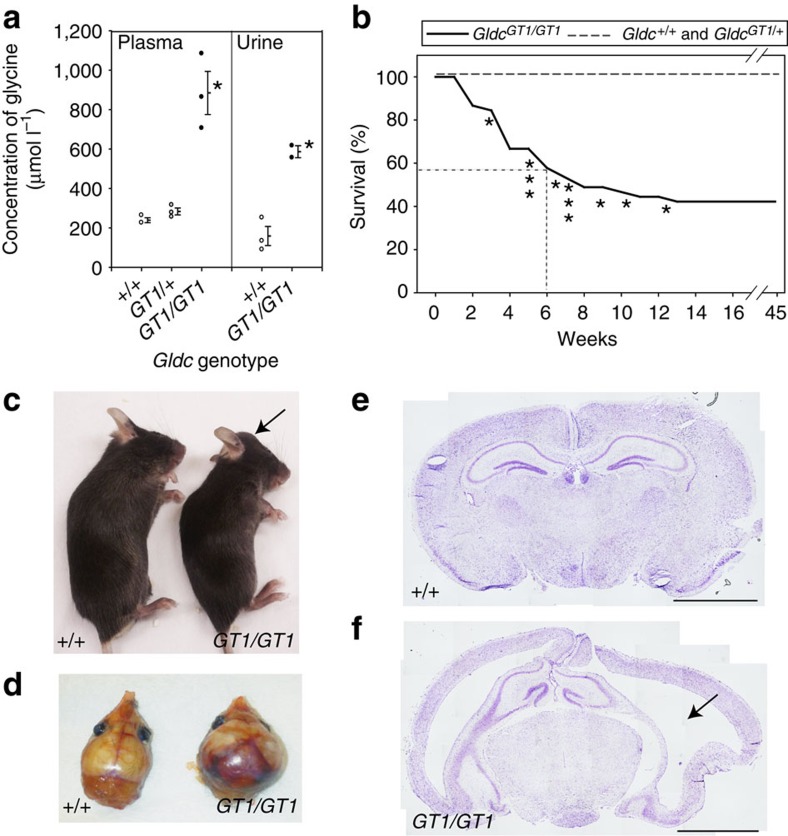
Elevated glycine, premature lethality and hydrocephalus. Concentrations of glycine in the plasma and urine (**a**; * significant difference from *Gldc*^*+/+*^, *P*<0.01 ANOVA). (**b**) Survival curve for *Gldc*^*GT1/GT1*^ (*n*=45). *Gldc*^*+/+*^ (*n*=10) and *Gldc*^*GT1/+*^ mice (*n*=6) up to 1 year of age. Approximately 22% of *Gldc*^*GT1/GT1*^ mice exhibited hydrocephalus (* indicates onset of hydrocephalus when mice were culled). In comparison with unaffected littermates, mice with hydrocephalus displayed a characteristic domed head (arrow in **c**), and expansion of the cranium with evident haemorrhage (**d**). Coronal sections at the level of the hippocampus (**e**,**f**) reveal enlargement of the lateral ventricles (arrow in **f**) in the brain of *Gldc*^*GT1/GT1*^ mice with the hydrocephalus (**e**,**f** are composite images of Cresyl violet (Nissl)-stained sections; scale bar, 2.5 mm).

The GCS provides the principal means for glycine catabolism and we observed a significant increase in plasma and urine glycine levels from *Gldc*^*GT1/GT1*^ mice compared with wild-type littermates, at 12–14 weeks of age (Fig. 2a). The mean glycine in plasma was 889 μM compared with 239 μM in wild types. This finding is consistent with the observation of elevated glycine levels in body fluids of NKH patients with deficient GCS function^7^.

Cohorts of *Gldc*^*GT1/GT1*^, *Gldc*^*GT1/+*^ and wild-type mice were monitored up to 12 months of age. We did not observe any obvious abnormalities among wild-type or heterozygous mice. However, *Gldc*^*GT1/GT1*^ mice exhibited premature lethality, with 55% dying between 2 and 12 weeks of age (Fig. 2b). Approximately half of these mice (22% of homozygous mutants) developed hydrocephalus, characterized by a dome-shaped appearance of the head (Fig. 2c) and enlargement of the cranium (Fig. 2d). Removal of the skull revealed swelling of the brain with evident haemorrhage and dramatic enlargement of the ventricles was apparent upon sectioning (Fig. 2e,f). These signs occurred at 3–12 weeks of age and progressed rapidly over an ~48-h period, at which time animals were killed due to obvious ill health. Homozygous mutant mice that survived to 16 weeks of age appeared normal to 1 year of age.

A review of NKH patients provided some evidence for a gender difference in severity, with greater survival and improved developmental progression among boys^7^. Among the cohort of post-natal *Gldc*^*GT1/GT1*^ mice monitored for survival, 65% (13/20) of males survived to 6 weeks of age compared with 33% (7/21) of females. Although this difference in survival was not statistically significant, it was notable that hydrocephalus occurred in 10/21 females but in 0/20 males (*P*<0.001; Fisher exact test). The hydrocephalus phenotype was not totally exclusive to females as occasional hydrocephalus has been observed among male homozygotes collected for metabolite/mRNA analysis.

### Brain abnormalities are present prenatally in Gldc mutants

**Figure 3:**
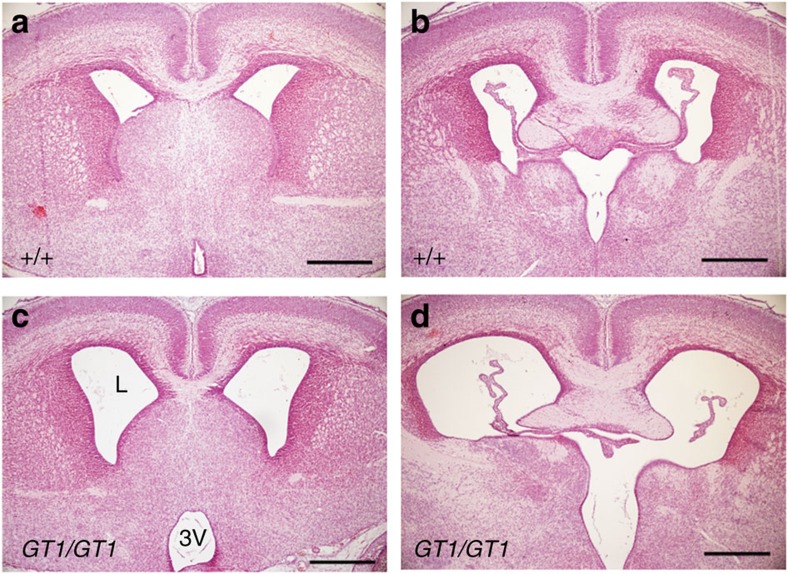
Abnormal brain phenotype is evident prenatally. Coronal sections through the brain of wild-type (**a**,**b**) and homozygous *Gldc*^*GT1*^ mutant (**c**,**d**) fetuses at E18.5 reveals enlargement of the lateral (L) and third ventricles (3V). Sections A and C are at a more anterior position than B and D; scale bar, 0.5 mm.

NKH presents in neonates; however, it is not clear whether Gldc activity is required in the fetus and to what extent the disease manifests prenatally^7^,^15^. In the *Gldc*^*GT1/GT1*^ mouse, analysis of tissue extracts from embryos at E11.5 showed that glycine levels were already significantly elevated by this stage of development (see below). In fact, analysis of litters collected at late gestation (E15.5 and E18.5) revealed two separate phenotypes. A proportion of *Gldc*^*GT1*^ embryos displayed exencephaly, a cranial NTD in which neural tissue bulges from the brain (see below). Among embryos that did not display exencephaly, histological analysis at E18.5 revealed striking enlargement of the brain ventricles among a proportion (two out of five) of homozygous *Gldc*^*GT1/GT1*^ mutants (Fig. 3c,d). Thus, structural abnormalities of the brain are already present prenatally among a subset of mice lacking GCS function.

### Gldc function is required for neural tube closure

**Figure 4:**
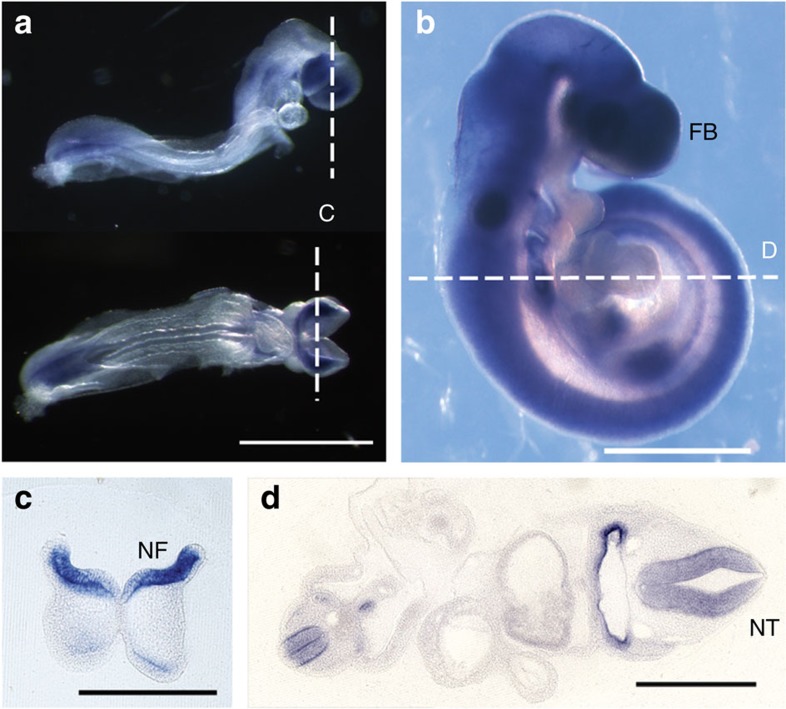
*Gldc* is expressed in the neuroepithelium. Whole mount *in situ* hybridization at E8.5 (**a**,**c**) and E9.5 (**b**,**d**) shows abundant expression in the neural folds (NFs) and neural tube (NT) with particularly intense staining in the forebrain (FB) at both stages. Dashed lines in **a**,**b** indicate level of sections in **c**,**d**, respectively. Expression analysed in at least six different embryos at each stage. Scale bar, 1 mm.

*Gldc* was previously found to be expressed in the CNS of rat embryos at late gestation (E15.5 and E18.5)^16^. In addition to the detection of elevated glycine at E11.5 (see below), the observation of NTDs among mutant embryos also suggested a function of Gldc earlier in development. Analysis of the spatiotemporal pattern of *Gldc* expression by whole mount *in situ* hybridization at the stage of neural tube closure revealed intense expression in the open neural folds of the prospective forebrain and posterior neuropore at E8.5 (Fig. 4a,c; Supplementary Fig. 1a,d–g) and throughout the closed neural tube at E9.5 (Fig. 4b,d). Specific expression of *Gldc* in the closing neural folds is consistent with the hypothesis that activity of this enzyme is required for neural tube closure. Expression persisted in the closed neural tube at E9.5 and E10.5, suggesting a continued requirement for GCS function in the CNS (Fig. 4, Supplementary Fig. 1).

**Figure 5:**
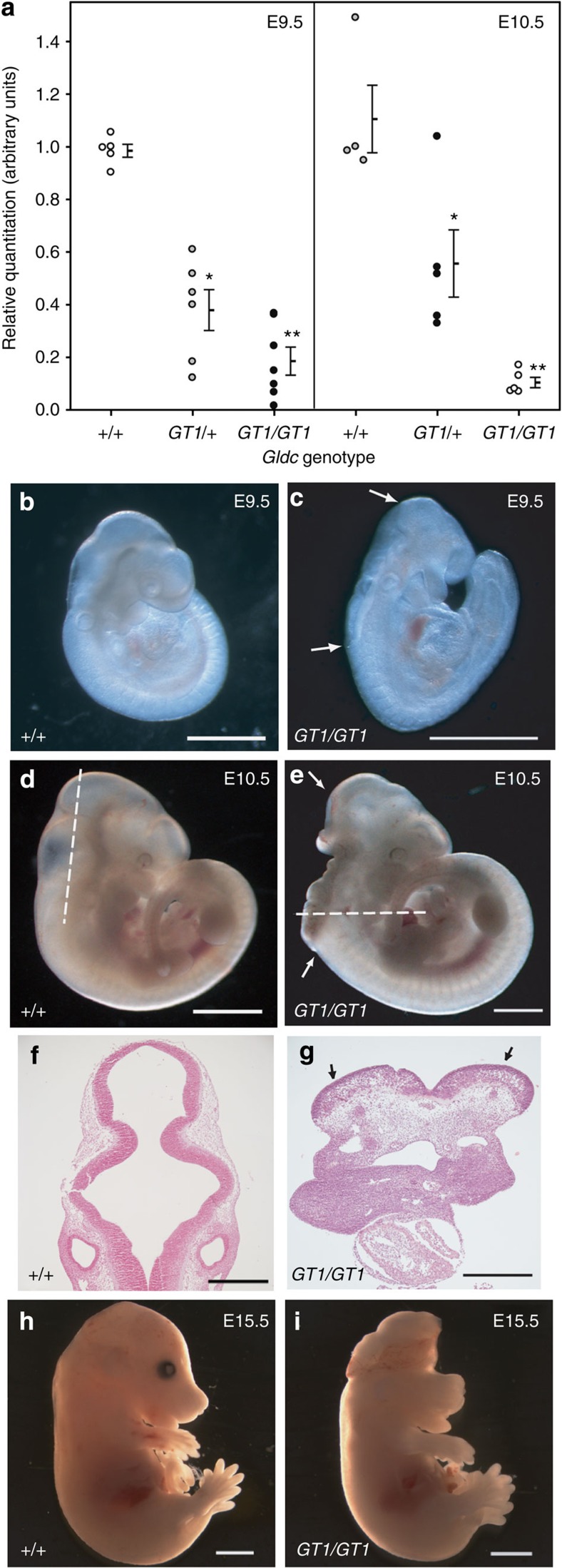
Reduced expression of *Gldc* causes NTDs. Abundance of *Gldc* mRNA (**a**) was diminished in *Gldc*^*GT1/GT1*^ compared with both wild-type and *Gldc*^*GT1/+*^ embryos at E9.5 and E10.5 (** significantly different to +/+ and *Gldc*^*GT1/+*^ *P*<0.001, ANOVA). *Gldc* expression in *Gldc*^*GT1/+*^ embryos at these stages was also significantly lower than in wild-types (*P<0.001, ANOVA). Exencephaly occurred among *Gldc*^*GT1/GT1*^ fetuses at E15.5 (**i**), but not in +/+ littermates (**h**). This phenotype results from failure of closure of the cranial neural folds at E9.5 (open region between arrows in **c**), whereas cranial closure is complete in *Gldc*^*+/+*^ embryos by this stage (**b**). The neural folds remain persistently open as evident in a transverse section (**g**) of a mutant embryo at E10.5 (**e**; black arrow indicates open neural folds). Sections in **f**,**g** are at the levels indicated in **d**,**e**. Scale bars, 1 mm (**b**–**e**), 2 mm (**f**,**g**) or 0.5 mm (**h**,**i**).

We generated homozygous *Gldc*^*GT1/GT1*^ embryos for more detailed analysis of the embryonic phenotype, particularly NTDs. Litters were collected at a series of developmental stages from E9.5 to E18.5. The numbers of embryos of differing genotypes (111+/+; 198 *Gldc*^*GT1/+*^; 115 *Gldc*^*GT1/GT1*^; Tables 1 and 2—untreated) did not differ from the predicted Mendelian ratio (*P*>0.05, *χ*^2^-test), indicating that the *Gldc*^*GT1*^ allele and loss of GCS function do not cause pre-neurulation embryonic lethality on a C57BL/6 genetic background. The efficacy of the gene-trap construct at neurulation stages was verified by quantitative real-time RT–PCR (qRT–PCR) on RNA samples isolated from litters of embryos at E9.5 and E10.5. In comparison with wild-type embryos, the abundance of *Gldc* mRNA was diminished, by 80–90% in homozygous mutant embryos and by 50–60% in heterozygotes (Fig. 5a). Thus, *Gldc*^*GT1*^ represents a strong hypomorphic allele.

As predicted from the observation of exencephaly (cranial NTDs) at later gestation (Fig. 5i), a proportion of homozygous mutant (*Gldc*^*GT1/GT1*^) embryos at E9.5–10.5 exhibited persistently open cranial neural folds, whereas the cranial neural tube was closed in stage-matched wild-type embryos (Fig. 5b–g). Among wild-type embryos at E9.5 the cranial neural tube was closed by the 16 somite stage, whereas the neural tube remained open among 29% of *Gldc*^*GT1/GT1*^ embryos with 18 somites or more, and were annotated as cranial NTDs (Table 1). Overall, open NTDs occurred in ~24% of *Gldc*^*GT1/GT1*^ embryos (Tables 1 and 2) and were also occasionally observed in heterozygous embryos (0.5%; 1 out of 198). Exencephaly results in death at, or shortly after, birth, which is reflected in the skewed Mendelian ratio that we previously observed at post-natal stages.

Female embryos have a greater susceptibility to cranial NTDs in humans, and this is also observed in a number of mouse NTD models^17^,^18^. However, among *Gldc*^*GT1/GT1*^ embryos for which sex was determined we observed almost identical frequency of NTDs among male (15 out of 85) and female (14 out of 78) homozygous mutant embryos.

### Impaired activity of Gldc disrupts FOCM

We investigated the mechanisms underlying Gldc-related NTDs. In addition to the accumulation of glycine, impaired GCS function is also predicted to lead to reduced supply of formate from mitochondrial FOCM. Quantification of plasma formate by gas chromatography mass spectrometry did not reveal significant variation between adult mice of different genotypes (Table 2). Thus, despite reduced glycine cleavage, formate production in *Gldc*-deficient mice was sufficient to at least maintain circulating levels.

**Figure 6:**
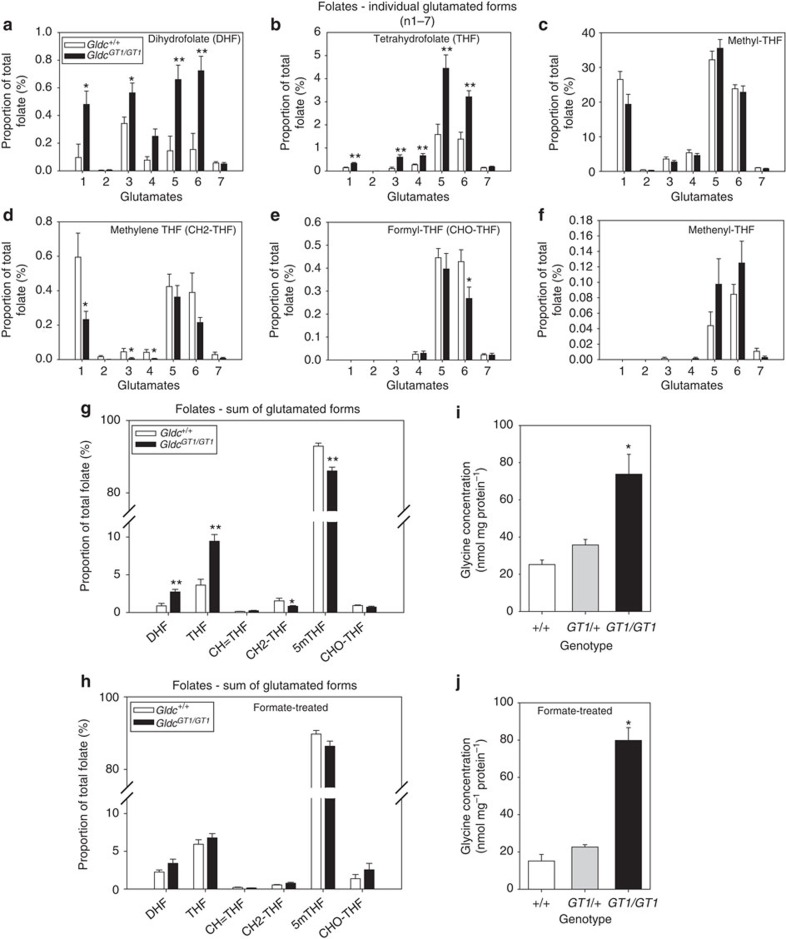
Elevated glycine and abnormal folate profile in *Gldc* mutant embryos. (**a**–**f**) Relative proportions (expressed as % of total folate) of mono- and polyglutamated forms of (**a**) DHF, (**b**) THF, (**c**) methyl-THF (5mTHF), (**d**) methylene-THF (CH_2_-THF), (**e**) formyl-THF (CHO-THF) and (**f**) methenyl-THF (CH=THF) in *Gldc*^*GT1/GT1*^ embryos (*n*=10) and *Gldc*^*+/+*^ (*n*=7) embryos at E11.5. (**g**) Summation of all glutamated forms (n1–7) for each folate. Asterisks indicate significant difference to wild-type (*P<0.05, **P<0.01; *t*-test). (**h**) There were no significant differences between formate-treated embryos of differing genotypes (*n*=8 of each genotype). Glycine content in untreated (**i**) and formate-treated (**j**) *Gldc*-deficient embryos at E11.5 (* significantly different from other genotypes, *P*<0.01 ANOVA).

In order to directly assess the effect of diminished GCS activity on cellular FOCM in the embryo, we analysed the folate profile of *Gldc* mutant and wild-type embryos at E11.5 using tandem mass spectrometry-based methodology that allows specific quantification of individual folates and their polyglutamated forms (1–7 glutamates) in single embryos. The overall folate profile was compared by summation of the proportions of individual glutamated forms (Fig. 6g). This analysis showed that the relative abundance of THF and dihydrofolate (DHF) were significantly higher in *Gldc*^*GT1/GT1*^ embryos than in wild types. In contrast, 5,10-methylene THF and 5-methyl THF were reduced in abundance, while formyl-THF showed a nonsignificant (*P*=0.052) trend towards reduced abundance.

Analysis of individual glutamated forms of each folate (Fig. 6a–f) showed corresponding changes to the overall summated data. Thus, DHF carrying 1, 3, 5 and 6 glutamates (glu 1, 3, 5, 6; Fig. 6a) and THF—glu 1, 3, 4, 5 and 6 (Fig. 6b) were significantly more enriched in *Gldc* mutants. The reduction in 5,10-methylene THF abundance principally corresponded to alteration of the glu1, 3 and 4 forms (Fig. 6d), while changes in individual glutamated forms of 5-methyl THF were not significant (Fig. 6c), although the overall abundance was diminished (Fig. 6g). The overall reduction in formyl-THF was not statistically significant (Fig. 6g, *P*=0.052); however, there was a significant reduction in the proportion of the glu6 form (Fig. 6e).

The alterations in the relative proportions of folates that we observed in *Gldc* mutant embryos are consistent with a paucity of one-carbon units being supplied from mitochondrial FOCM. Thus, the abundance of one-carbon-carrying ‘activated’ folates (methylene, formyl and methyl THF) was diminished, while ‘non-activated’ folates (DHF and THF) increased. The changes were also in line with predictions from computational modelling of hepatic FOCM^19^.

### NTDs result from reduced supply of one-carbon units

Impairment of mitochondrial FOCM owing to Gldc deficiency appears to reduce the supply of one-carbon units. We tested the effect of simultaneously depleting the abundance of folate available from the diet. Female mice were maintained on a folate-deficient diet, with antibiotics to remove folate-synthesizing gut microflora, before experimental mating and until collection of litters. We previously found that this regime significantly reduces maternal blood folate concentration and embryonic folate content and increases the incidence of cranial NTDs in *Pax3*^*Sp*^ (*splotch*; *Sp*^*2H*^) and *Grhl3*^*ct*^ (*curly tail*) mutant mice^20^,^21^. In contrast, NTDs did not occur at increased frequency among folate-deficient *Gldc* mutant mice (Table 3). Thus, it appears that generation of THF did not become limiting under folate-deficient conditions such that sufficient THF was available to carry available formate. Interestingly, maintenance on a folate-deficient diet resulted in a significant increase in circulating formate in *Gldc*^*GT1/+*^ dams (Table 3), suggesting that one-carbon units had ‘saturated’ available folate and were released into the circulation.

The redistribution of folates identified by folate profiling showed that *Gldc* mutation was sufficient to alter FOCM, and the changes in relative abundance of specific folates are consistent with a reduced flux of one-carbon units into the folate cycle. We hypothesized that diminished production of formate could cause NTDs. In order to enhance the supply of one-carbon units, embryos were supplemented with sodium formate in the drinking water of pregnant dams from the first day of pregnancy. This treatment resulted in elevated maternal circulating formate levels and striking prevention of NTDs in the offspring (Table 3). Glycine levels were elevated at E11.5 in *Gldc* mutant embryos (Fig. 6i) and were not corrected by formate treatment (Fig. 6j). However, in parallel with the rescue of NTDs, the folate profile of *Gldc*^*GT1/GT1*^ embryos was normalized, such that they were indistinguishable from wild-type formate-treated littermates (Fig. 6h). Thus, in homozygous *Gldc* mutant embryos, formate treatment led to a reduction in the level of THF and an increase in the abundance of formyl THF. Consistent with these findings, we did not detect significant differences in the proportion of individual glutamated folates in formate-treated *Gldc* mutant and wild-type embryos (Supplementary Fig. 2).

**Figure 7:**
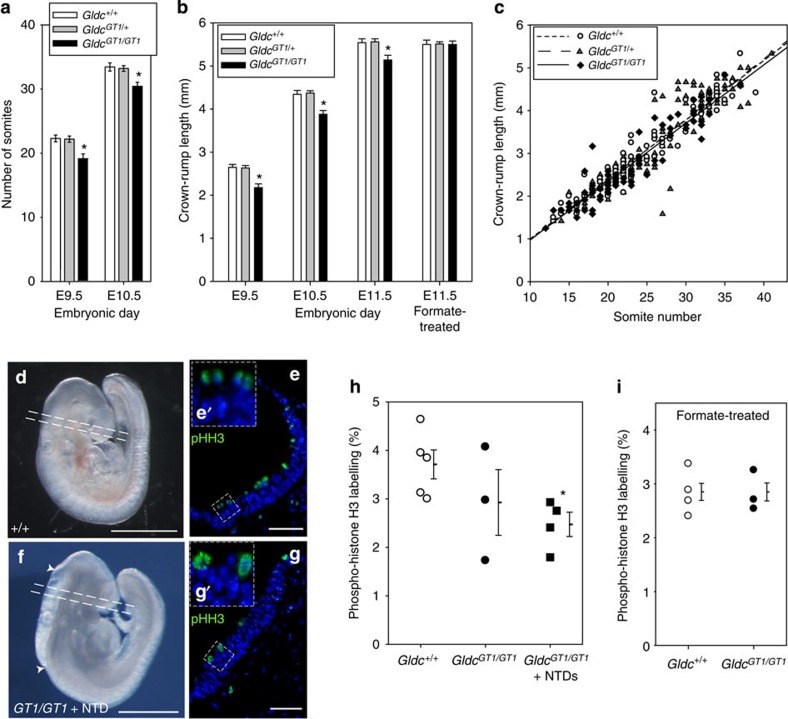
*Gldc* deficiency causes growth retardation that is normalized by formate supplementation. (**a**) Number of somites and (**b**) crown-rump length of neurulation-stage embryos (* indicates significant difference to other genotypes at the same stage; (**a**) *P*<0.01, (**b**) *P*<0.001, ANOVA; *n*=28–64 embryos of each genotype per stage). (**c**) Crown-rump length increases with somite number at a similar rate in all genotypes as indicated by the similar slopes of the regression lines in plots of crown-rump length against somite number for embryos with 12 or more somites (when turning has occurred). (**d**–**i**) Cellular proliferation in the neural folds at E9.5 in +/+ (**d**,**e**) and *Gldc*^*GT1/GT1*^ (**f**,**g**) embryos, matched for somite stage. Sections at the level of the rostral hindbrain (region indicated by dotted lines in **d**,**f**) immunostained for the mitosis marker phospho-histone H3 (pHH3; four to six sections analysed per embryo). Representative sections (**e**,**g**) show the typical apical location of mitotic nuclei (enlargements of the boxed regions are shown in **e**′ and **g**′; pHH3 staining is green, DAPI-stained nuclei are blue). Scale bar, 50 μm. (**h**,**i**) Phospho-histone H3 labelling index among (**h**) untreated (* indicates significant difference from +/+, *P*<0.01) and (**i**) formate-treated embryos.

One-carbon units in FOCM are utilized for several downstream outputs including nucleotide biosynthesis, required for cell proliferation. We analysed parameters of developmental progression (somite number) and growth (crown-rump length) at stages during and immediately after neural tube closure. In comparison with wild types and heterozygotes, homozygous *Gldc*^*GT1*^ mutants had significantly fewer somites at E9.5 and E10.5 (Fig. 7a). At both stages, mutants had approximately three somites less than embryos of the other genotypes, equivalent to a developmental delay of around 6 h. The mean crown-rump length of *Gldc*-deficient embryos was smaller at each stage (Fig. 7b), indicative of growth retardation. Plots of crown-rump against somite number for individual embryos do not show any difference between genotypes (Fig. 7c), showing that *Gldc*^*GT1/GT1*^ embryos are the appropriate size for stage. Hence, GCS deficiency results in developmental delay but the relationship between growth and developmental progression is not disrupted.

NTDs result from the failure of closure of the neural folds (Fig. 5) and the neuroepithelium is a site of intense *Gldc* expression (Fig. 4). We therefore investigated whether cell proliferation was compromised in the neuroepithelium at the level of the rostral hindbrain, the region most frequently affected in *Gldc*^*GT1/GT1*^ embryos that developed NTDs (Fig. 7d–i). At E9.5, immediately after closure was complete in wild-type embryos, the labelling index for phospho-histone H3 (a marker of G2/M phase) was significantly lower, indicating a reduced rate of mitosis, in the neural folds of *Gldc*^*GT1/GT1*^ in which closure was incomplete (with NTDs; Fig. 7h). Among *Gldc*^*GT1/GT1*^ embryos in which cranial neural tube closure had achieved completion (without NTDs), the labelling index showed a greater range of values and did not differ significantly from either +/+ or *Gldc*^*GT1/GT1*^ embryos with NTDs (Fig. 7h).

We examined whether formate treatment corrected growth parameters in parallel with normalization of the folate profile. No genotype difference in crown-rump length was present among formate-treated litters, indicating that treatment enhanced the growth of *Gldc*-deficient embryos in parallel with prevention of NTDs (Fig. 7c). Consistent with these findings, analysis of the mitotic index in the neuroepithelium of formate-treated embryos showed that proliferation was enhanced in *Gldc*-deficient embryos, such that there was no significant difference between +/+ or *Gldc*^*GT1/GT1*^ embryos (Fig. 7i).

## Discussion

Gldc is an atypical PLP-dependent amino-acid decarboxylase in that the aminomethyl moiety of glycine is not released as methylamine but transferred to Gcsh^1^. Our findings highlight the significance of this mechanism in that both functions of Gldc, glycine decarboxylation and transfer of a one-carbon group into FOCM, appear to be essential for normal CNS development. Moreover, loss of each function may result in different phenotypes. Thus, we found that reduced expression of Gldc causes two distinct disorders in mice, failure of neural tube closure (NTDs) and NKH-like phenotypes.

Our findings in *Gldc* mutant mice clarify key questions about the relationship between NKH and NTDs in humans. Homozygous or compound heterozygous mutation of *GLDC* and *AMT* is well established as a cause of NKH^8^. In addition, heterozygous mutations of *AMT* or *GLDC* were identified in patients with NTDs^14^. These include mutations that confer reduced enzyme activity and were absent in controls, single-nucleotide polymorphism databases and the ExAC database of more than 60,000 unrelated individuals^22^. Given the estimated carrier frequency of NKH-causing mutations and the incidence of variants among NTD patients, it was estimated that carriers are at 10-fold increased risk of NTDs^14^. This hypothesis is consistent with the association of abnormal folate metabolism with susceptibility to these birth defects and was supported by the finding that *Amt* null mice develop NTDs at high frequency^14^. However, it remained possible that *Gldc* is not required for neural tube closure and/or that impaired GCS function could have a different outcome in humans and mice such that NKH phenotypes would not be observed in mice.

In the current study, we were able to address this issue by analysis of *Gldc* loss-of-function mice, in which partial penetrance of NTDs provided an opportunity to examine late fetal and post-natal phenotypes. The presence of cranial NTDs in *Gldc* mutants demonstrates a necessary role for glycine decarboxylation in neural tube closure confirming the requirement for GCS activity in this process. Neural tube closure occurs at E8.5–10.5 in mice and weeks 3–4 of human pregnancy^10^. At post-natal stages, *Gldc* mice that did not succumb to NTDs exhibited accumulation of glycine in body fluids, which is the characteristic hallmark of NKH. Hence, *Gldc* is a gene whose mutation can result in two different diseases.

In homozygous *Gldc* mutant mice, the observed three- to fourfold increase in concentration of plasma glycine is comparable to that reported in NKH patients^8^,^15^,^23^,^24^,^25^. Loss of *Gldc* function also resulted in premature lethality and prominent hydrocephalus in some individuals. Acquired hydrocephalus with dilatation of the ventricles is a feature of NKH^7^,^15^,^26^,^27^. The observation of an analogous phenotype in mice indicates that this is a primary feature of NKH rather than being linked to an NKH-related therapeutic. A survey of clinical outcome among 65 NKH patients previously suggested that the disease course may be more severe among females^7^. Our analysis of post-natal *Gldc* mutant mice showed a higher rate of hydrocephalus among females, with a correspondingly lower survival rate, supporting the hypothesis that gender differences may be present in NKH. In contrast to the greater predisposition of female *Gldc* mutant mice to hydrocephalus, there was no sex bias in the development of NTDs.

The typical neonatal presentation of NKH patients, with evident neurological signs within the first few days of life, suggests that pathogenesis may commence before birth. Increased concentration of glycine in cerebrospinal fluid has been reported at birth, suggesting that accumulation may begin during fetal life^28^. Our findings support this hypothesis; in rodents GCS-encoding genes are expressed in the CNS as early as neurulation stages as well as during later fetal stages^16^. Moreover, analysis of tissue extracts showed that glycine is already present at increased levels in *Gldc* mutant embryos by E11.5 of mouse gestation. Brain malformations may also begin to develop before birth in NKH patients. For example, hypoplasia of the corpus callosum has been reported at prenatal as well as neonatal stages^29^,^30^. In *Gldc*-deficient mice, structural abnormalities were also present at fetal stages, with enlargement of the ventricles, a precursor of hydrocephalus, detectable in some homozygous mutants. This phenotype was present in a distinct group of fetuses from those that developed NTDs, in which the open brain precludes development of hydrocephalus.

Neurological signs among NKH patients have principally been attributed to the accumulation of glycine, acting through inhibitory glycine receptors and as co-agonist for glutamate at NMDA (*N*-methyl-d-aspartate) receptors^7^,^15^. In contrast, the mechanisms underlying GCS-related NTDs have not been defined. Our data show that in addition to cleavage of glycine, Gldc activity also plays a key role in FOCM. Mitochondrial FOCM supplies ~80% of one-carbon units that flow through cytoplasmic FOCM into the methylation cycle^31^. Mitochondrial FOCM generates formate, with the principal one-carbon donors being serine (catalysed by Shmt2) or glycine (mediated by the GCS) with additional contribution from dimethylglycine and sarcosine^2^,^32^. As plasma formate was unaffected in adult *Gldc* mutant mice, loss of GCS activity does not diminish production of formate to the extent that circulating levels are compromised. However, despite the possible supply of one-carbon units from sources other than glycine, loss of Gldc function in the embryo had significant effects on cellular folate profiles.

The alterations in the relative proportions of folates observed in *Gldc* mutant embryos are consistent with a paucity of one-carbon units being supplied from mitochondrial FOCM. Thus, the abundance of one-carbon-carrying ‘activated’ folates (methylene, formyl and methyl THF) was diminished, while ‘non-activated’ folates DHF and THF increased. Rescue of NTDs by formate supplementation suggests that it is the lack of one-carbon units, rather than excess glycine, which is responsible for failure of neural tube closure in *Gldc*-deficient embryos. Consistent with this idea, formate treatment normalized the folate profile of *Gldc* mutants but not glycine levels. These findings also correlate with the occurrence of NTDs in gene-trap mutants of *Mthfd1L*, which catalyses the final step in production of formate in the mitochondria^33^. Overall, these findings reinforce the view that neural tube closure is crucially dependent on the integrity of mitochondrial FOCM.

Interestingly, folate deficiency did not exacerbate neural tube closure in *Gldc* mutants, unlike in *Pax3*, *Grhl3*^*ct*^ and *Shmt1* mutant mice^20^,^21^,^34^, suggesting that abundance of the THF one-carbon-carrying ‘backbone’ did not become limiting in the context of reduced formate supply from mitochondrial FOCM. This observation highlights the potential importance of differential effects of supplemental folate-related molecules for prevention of NTDs. For example, the most widely utilized supplement, folic acid, enters the folate cycle as DHF and does not carry a one-carbon group, and thus is dependent on sufficient endogenous supply of one-carbon groups to be effective.

What is the requirement for FOCM in neural tube closure? Reduced flux through FOCM could result in diminished methylation and/or impairment of nucleotide biosynthesis, availability of folate being essential for embryonic growth and developmental progression^20^. The one-carbon donors for these reactions (Fig. 1a), methyl THF (methylation), methylene THF (dTMP synthesis) and formyl THF (purine synthesis), were all depleted in Gldc-deficient embryos suggesting that either or both of these outputs could be compromised. Inhibition of the methylation cycle and/or DNA methylation causes NTDs in mouse embryos^35^,^36^. Impaired nucleotide biosynthesis is also associated with development of NTDs^34^,^37^, while supplementation with nucleotides may normalize neural tube closure in some NTD models^38^. *Gldc*-deficient embryos showed delays in developmental progression and growth. Moreover, there was a lower rate of cellular proliferation in the cranial neural folds of embryos with NTDs, a site of abundant *Gldc* expression in our expression analysis. Growth of the embryos and proliferation in the neural folds were both normalized by formate treatment in parallel with prevention of NTDs. Overall, both reduced growth and a lower rate of neuroepithelial proliferation correlated with failure of cranial neural tube closure. Together, these findings support the hypothesis that suppression of FOCM, resulting from *Gldc* mutation, may disrupt neural tube closure through impairment of the methylation cycle and/or inadequate nucleotide biosynthesis, leading to reduced cell proliferation.

## Methods

### Gldc mutant mice

In order to generate mice carrying a gene-trap allele of *Gldc* (here denoted *Gldc*^*GT1*^), we obtained the EUCOMM, EUCG0001_D02 embryonic stem cell line from the European Mouse Mutant Cell Repository. Chimeric mice carrying the *Gldc* gene-trap allele were generated by blastocyst injection of ES cells (Embryonic Stem Cell Facility, UCL Institute of Child Health), and mated to 129/Sv mice to test for germline transmission and establish a colony of heterozygous mice. Preliminary matings revealed a high rate of resorption as well as severe developmental retardation of the few homozygous mutant embryos that could be obtained. Subsequently, the *Gldc* gene-trap allele was crossed on the C57Bl/6 background for five generations in order to generate mice for experimental matings. Experimental litters were generated by heterozygous matings. In addition, viable homozygous mutant mice were intercrossed to generate litters of *Gldc*^*GT1/GT1*^ mice for the survival study (Fig. 2b). Animal studies were carried out under regulations of the Animals (Scientific Procedures) Act 1986 of the UK Government, and in accordance with the guidance issued by the Medical Research Council, UK in *Responsibility in the Use of Animals for Medical Research* (July 1993).

Formate treatment was performed by the addition of sodium formate (30 mg ml^−l^) to the drinking water of females from the first day of pregnancy. For dietary experiments, female mice were maintained on a folate-deficient diet containing 1% succinyl sulfathiazole for 3 weeks before mating and during pregnancy. This diet produces significant reduction in maternal serum folate and elevation of plasma homocysteine^20^.

### Collection of embryos and histology

Experimental litters were generated by timed matings: noon on the day of finding a copulation plug was designated 0.5 days of embryonic development (E0.5). Pregnant females were killed by cervical dislocation and embryos were dissected from the uterus in Dulbecco’s Modified Eagle’s Medium (Invitrogen) containing 10% fetal calf serum (Sigma). Measurements were made with an eyepiece graticule. Scoring of NTDs and metabolite assays were performed blind to genotype. Embryos or fetuses were rinsed in PBS and stored at −80 °C (for RNA and metabolite analysis) or fixed in 4% paraformaldehyde (PFA) in PBS at 4 °C overnight (for histology and *in situ* hybridization). For histological analysis, PFA-fixed embryos and brains of 5-week-old mice were dehydrated, embedded in paraffin wax and sectioned at 7-μm thickness. Sections were stained with haematoxylin/eosin and cresyl violet for embryo and brain samples, respectively.

### Immunohistochemistry

Proliferation analysis was carried out by immunostaining for phosphohistone H3 on 4-μm transverse sections at the axial level of the closing neural folds in the rostral hindbrain in somite-matched embryos at E9.5 (18–24 somites). Primary and secondary antibodies were anti-phospho-histone H3 (1:250, Millipore) and Alexa Fluor 488-conjugated anti-rabbit (1:500, Invitrogen). For nuclear staining, cells were incubated with 4,6-diamidino-2-phenylindole (DAPI, 1:10,000 in PBS). Fluorescent images were collected on an Axiophot microscope (Zeiss) with a DC500 camera (Leica), using FireCam software (Leica). Images were analysed using the Cell Counter plugin of the Image J software (US National Institutes of Health, Bethesda, MD, USA). Cells in mitosis were scored by visual inspection of pH3-positive cells. Total cell number (visualized by DAPI staining) in the neural folds was counted for normalization of phospho-histone H3 counts.

### Sequencing and genotyping

The efficacy of gene trapping was initially tested by X-Gal staining of ES cells and heterozygous embryos on the basis of the reported presence of a *β-geo* reporter in the trapping cassette. However, as staining was negative, the insertion site and structure of the gene-trap cassette were verified by PCR amplification (long-range PCR) and sequencing of genomic DNA. We confirmed the presence of the gene-trap cassette inserted at nucleotide 30,171,315 in intron 2. This included a neomycin selection cassette as well as Frt, F3, loxP and lox5171 sites. The β-geo reporter is not present.

Embryos and mice were genotyped by PCR amplification of genomic DNA. Intron-specific primers were used to amplify a 644-bp product from wild-type and heterozygous samples, while use of an intron-specific primer with a gene-trap-specific primer generated a 602-bp product corresponding to the trapped allele. Embryonic sex was determined by PCR using specific primers to amplify the *Smcx* and *Smcy* genes (5′- CCGCTGCCAAATTCTTTGG -3′ and 5′- TGAAGCTTTTGGCTTTGAG -3′), which give a single band in females and two bands in males^20^.

### qRT–PCR

RNA was prepared using TRI reagent (Invitrogen), genomic DNA removed by DNase I digestion (DNA-free, Ambion) and first strand cDNA synthesis performed using random hexamers (Superscript VILO cDNA synthesis kit). The abundance of *Gldc* mRNA was analysed using qRT–PCR (MESA Blue Mastermix for SYBR Assay, Eurogentec) on a 7500 Fast Real Time PCR system (Applied Biosystems), with each sample analysed in triplicate. Primers were located in exons 2 and 4 (5′- AGCATTGATGAGCTCATCGAG -3′ and 5′- TCCAGCAGGGAAGCGTTGGC -3′) of mouse *Gldc* to amplify the wild-type but not the mutant transcript. Results were normalized to abundance of *gapdh* mRNA.

### Enzymatic assay

Activity of the GCS was measured in homogenized liver samples by determination of decarboxylation of [C^14^] glycine in the presence of cofactors (THF, pyridoxal phosphate and NAD)^39^. Liver samples were homogenized, on ice, in cofactor solution (0.1 M Tris-Cl, pH 8.0, pyridoxal phosphate (0.4 mg ml^−l^), dithiothreitol (0.8 mg ml^−1^) and NAD (3 mg ml^−1^)). THF (5 mg ml^−1^) was added to aliquots, in triplicate, and 5 μl glycine added (two parts unlabelled 80 mM glycine: 1 part [1-^14^C] glycine 0.1 mCi ml^−1^, Perkin Elmer). Samples were incubated for 2 h at 37 °C in sealed plastic tubes with a square of filter paper, soaked in 1 M NaOH and suspended from the lid. The reaction was terminated by addition of 30% TCA and the amount of ^14^CO_2_ trapped on the filter paper measured in a scintillation counter. Total protein content of an aliquot of the homogenates was measured using a protein assay kit (Perbio).

### Whole mount *in situ* hybridization

A 587-bp region corresponding to nucleotides 30,151,484–30,139,376 of the *Gldc* transcript (ENSMUST00000025778) was cloned by RT–PCR into pGEM-T (Promega) and used to generate a digoxygenin-labelled cRNA probe by reverse transcription. Primers for RT–PCR were 5′- GCCAACGCTTCCCTGCTGGA -3′ and 5′- AGGGCCTGAGCCGTGCAGAT -3′, located in exon 4 and 8/9 (primer spans intron–exon boundary). Embryos were rehydrated and washed in PBS containing 0.1% Tween-20 (PBT), bleached with 6% hydrogen peroxide and treated with 5 μg ml^−1^ proteinase K followed by 2 mg ml^−1^ glycine, and then re-fixed in 0.2% gluteraldehyde prepared in 4% PFA. After fixation, embryos were washed in PBT and incubated in pre-hybridization buffer containing 50% formamide, 5 × saline sodium citrate at pH 4.5, 50 μg ml^−1^ yeast RNA, 1% sodium dodecyl sulphate and 50 μg ml^−1^ heparin in DEPC-treated water. The mRNA probe was added hybridized overnight at 70 °C. Embryos were washed in solution I (50% formamide, 5 × SSC and 1% SDS) at 70 °C, followed by solution II (50% formamide, 2 × SSC and 1% SDS) at 65 °C and TBST at room temperature. Embryos were blocked with 10% sheep serum, and then incubated overnight with anti-Digoxigenin-AP antibody (1:2,000, Roche) in 1% sheep serum. Colour detection was carried out using NBT/BCIP developing solution (Roche) in NTMT (100 mM sodium chloride, 100 mM Tris (pH 9.5), 50 mM magnesium chloride, 1% Tween-20). After colour development, embryos were photographed with a DFC490 camera (Leica) mounted on a Stemi SV11 stereomicroscope (Zeiss), and then embedded in gelatin/albumin medium set with 0.2% gluteraldehyde. Sections of 50-μm thickness was made with a vibratome, mounted with glycerol and photographed with an Axiophot microscope (Zeiss).

### Metabolite analysis

Blood was collected by terminal cardiac exsanguination, transferred to lithium-heparin tubes (BD Microtainer) and immediately centrifuged for the isolation of plasma. Embryo and tissue extracts were prepared by homogenization of samples by sonication in (i) PBS containing 1 × protease inhibitor cocktail (Roche; for amino-acid assay) or (ii) buffer containing 20 mM ammonia acetate, 0.1% ascorbic acid, 0.1% citric acid and 100 mM dithiothreitol at pH 7 (for mass spectrometry). An aliquot was removed for protein determination by the Bradford assay. Protein was removed by precipitation by addition of two volumes of acetonitrile and centrifugation (12,000 *g* at 4 °C). Supernatants were transferred, lyophilized, stored at −80 °C and re-suspended in dH_2_O before analysis.

*Glycine*. Analysis was performed in the Chemical Pathology Department of Great Ormond Street Hospital for Children NHS Foundation Trust using an established method for amino-acid analysis by ion-exchange chromatography. Protein was precipitated by the addition of 5-sulphosalicylic acid containing a specified quantity of internal standard (S-(2-Aminoethyl)-L-cysteine hydrochloride), in a ratio of 2:1 (sample:internal standard solution). Samples were precipitated for 30 min at 4 °C, centrifuged and the supernatant removed and filtered before analysis. The sample (60 μl) was injected on a Lithium High Performance Physiological Column (cation exchange) on a Biochrom 30 amino-acid analyser. Amino acids were detected spectrophotometrically at 570 nm by post-column derivatization with ninhydrin. For embryos, concentration was normalized to protein content.

*Formate*. Formate was derivatized with pentofluorobenzyl bromide and assayed by gas chromatography mass spectrometry^40^. Alkylation was carried out by mixing 10 μl of internal standard solution (10 ml 0.5 M phosphate buffer (pH 8.0) plus 3 ml of internal standard (1 mM sodium ^13^C-formate in HPLC-grade water solution), 25 μl of sample or standard and 65 μl of 100 mM pentafluorobenzyl bromide (in acetone), vortexing for 1 min and incubation for 15 min at 60 °C. Tubes were cooled to room temperature; 165 μl of *n*-hexane was added and vortexed for 1 min after which two layers are separated. The organic phase (100 μl; top layer) was transferred to glass inserts (Thermo Fisher no. 200-670) and analysed by gas chromatography–mass spectrometry (GC–MS) on a Thermo Fisher TRACE GC ULTRA equipped with an ISQ mass selective detector and an AS3000 auto-sampler. The GC separation was carried out by injecting a 1-μl aliquot of the organic phase on an Agilent DB-225J&W column under the following conditions: carrier gas, He; gas flow, 1.5 ml min^−1^; inlet temperature, 220 °C; transfer line temperature, 300 °C; ion source temperature, 300 °C. The GC temperature programme was initiated at a column temperature of 50 °C for 2 min, followed by an increasing gradient of 30 °C min^−1^ to a final temperature of 220 °C. MS was carried out in the electron-impact ionization mode, monitoring *m/z* at 226 and 227 (representing, respectively, the PFBBr (pentafluorobenzyl bromide) derivatives of ^12^C-formate and ^13^C-formate). Results were quantified by reference to a standard curve composed of 0, 25, 50, 75 and 100 μM formate, plotted against the ^12^C/^13^C-formate ratio.

*Folate mass spectrometry*. Folate analysis was carried out by ultraperformance liquid chromatography tandem mass spectrometry (UPLC-MS/MS) as described previously^41^,^42^, with adaptations. Lyophilized samples were resuspended in 30 μl water (milli-Q) and centrifuged for 5 min at 12,000 × *g* at 4 °C. Metabolites were resolved by reversed-phase UPLC (Acquity UPLC BEH C18 column, Waters Corporation, UK). Solvents for UPLC were as follows: Buffer A, 5% methanol, 95% Milli-Q water and 5 mM dimethylhexylamine at pH 8.0; Buffer B, 100% methanol, 5 mM dimethylhexylamine. The column was equilibrated with 95% Buffer A: 5% Buffer B. The sample injection volume was 25 μl. The UPLC protocol consisted of 95% Buffer A: 5% Buffer B for 1 min, followed by a gradient of 5–60% Buffer B over 9 min and then 100% Buffer B for 6 min before re-equilibration for 4 min. The metabolites were eluted at a flow rate of 500 nl min^−1^ and the wash step with 100% Buffer B was at flow rate of 600 nl min^−1^. The UPLC was coupled to a XEVO-TQS mass spectrometer (Waters Corporation) operating in negative-ion mode using the following settings: capillary 2.5 kV, source temperature 150 °C, desolvation temperature 600 °C, cone gas flow rate 150 l h^−,^ and desolvation gas flow rate 1,200 l h^−1^. Folates were measured by multiple reaction monitoring with optimized cone voltage and collision energy for precursor and product ions (Supplementary Table 1). Analysis of standard solutions indicates that sample preparation at pH 7 (optimal for detection of all six folate species) may result in conversion of up to 20% of CH_2_-THF to THF. In order to minimize variation in sample or running conditions between genotypes, samples of each genotype were analysed alternately.

## Additional information

**How to cite this article:** Pai, Y. J. *et al*. Glycine decarboxylase deficiency causes neural tube defects and features of non-ketotic hyperglycinemia in mice. *Nat. Commun.* 6:6388 doi: 10.1038/ncomms7388 (2015).

## Supplementary Material

Supplementary Information Supplementary Figures 1-2 and Supplementary Table 1.

## Figures and Tables

**Table 1:** Frequency of NTDs among *Gldc* ^*GT1*^ litters.

**Stage**	**Total**	** Gldc ** ^**+/+**^		** Gldc ** ^**GT1/+**^		** Gldc ** ^**GT1/GT1**^	
		***n***	**NTDs**	***n***	**NTDs**	***n***	**NTDs**
E9.5	159	45	0 (0%)	76	0 (0%)	38	11 (29%)*
E15.5–E18.5	146	40	0 (0%)	62	0 (0%)	44	11 (25%)*

NTD, neural tube defect.

At E9.5 cranial NTDs were defined as open neural folds among embryos with 18 or more somites. Litters (22–24) were analysed at each developmental stage. In *Gldc^GT1/GT1^* NTDs occur at significantly higher frequency than among other genotypes (*P<0.001; *z*-test).

**Table 2:** Plasma formate concentration in female mice.

**Genotype**	***n***	**Concentration of formate (μM)**
* Gldc * ^*+/+*^	3	27.7±1.9
* Gldc * ^*GT1/+*^	3	27.7±1.6
* Gldc * ^*GT1/GT1*^	2	27.9±3.1

**Table 3:** Formate supplementation of *Gldc* ^*GT1*^ litters.

**Treatment**	**Total**	** Gldc ** ^**+/+**^		** Gldc ** ^**GT1/+**^		** Gldc ** ^**GT1/GT1**^		**Maternal Gldc**^**GT1/+**^ **plasma formate (μM)**
		***n***	**NTDs**	***n***	**NTDs**	***n***	**NTDs**	
Untreated	109	16	0 (0%)	60	1 (1.7%)	33	6 (18%)	28±2
Formate	97	25	0 (0%)	49	0 (0%)	23	0 (0%)	431±250
Folate-deficient	70	18	0 (0%)	28	0 (0%)	24	4 (17%)	52.0±7*

ANOVA, analysis of variance; NTD, neural tube defect.

Pregnant dams were treated with sodium formate in drinking water (30 mg ml^−1^). No NTDs were observed among embryos analysed at E11.5. Numbers of litters analysed were 14 controls (E11.5), 14 formate-treated (E11.5) and 12 folate-deficient (E10.5–11.5). Maternal formate (*n*=3 *Gldc*^*GT1/+*^ mice of each treatment group) was at least sixfold (6- to 30-fold) higher than among untreated mice. Folate deficiency results in a significant increase in circulating formate levels (*P<0.05; ANOVA). Values are given as mean±s.e.m.
