# Supplementary material for: Glycine decarboxylase deficiency causes neural tube defects and features of non-ketotic hyperglycinemia in mice
Source: Nat Commun. 2015 Mar 4;6:6388. doi: 10.1038/ncomms7388 (PMC4366506; doi:10.1038/ncomms7388)
Supplement: Supplementary Information — Supplementary Figures 1-2 and Supplementary Table 1. [file ncomms7388-s1.pdf]

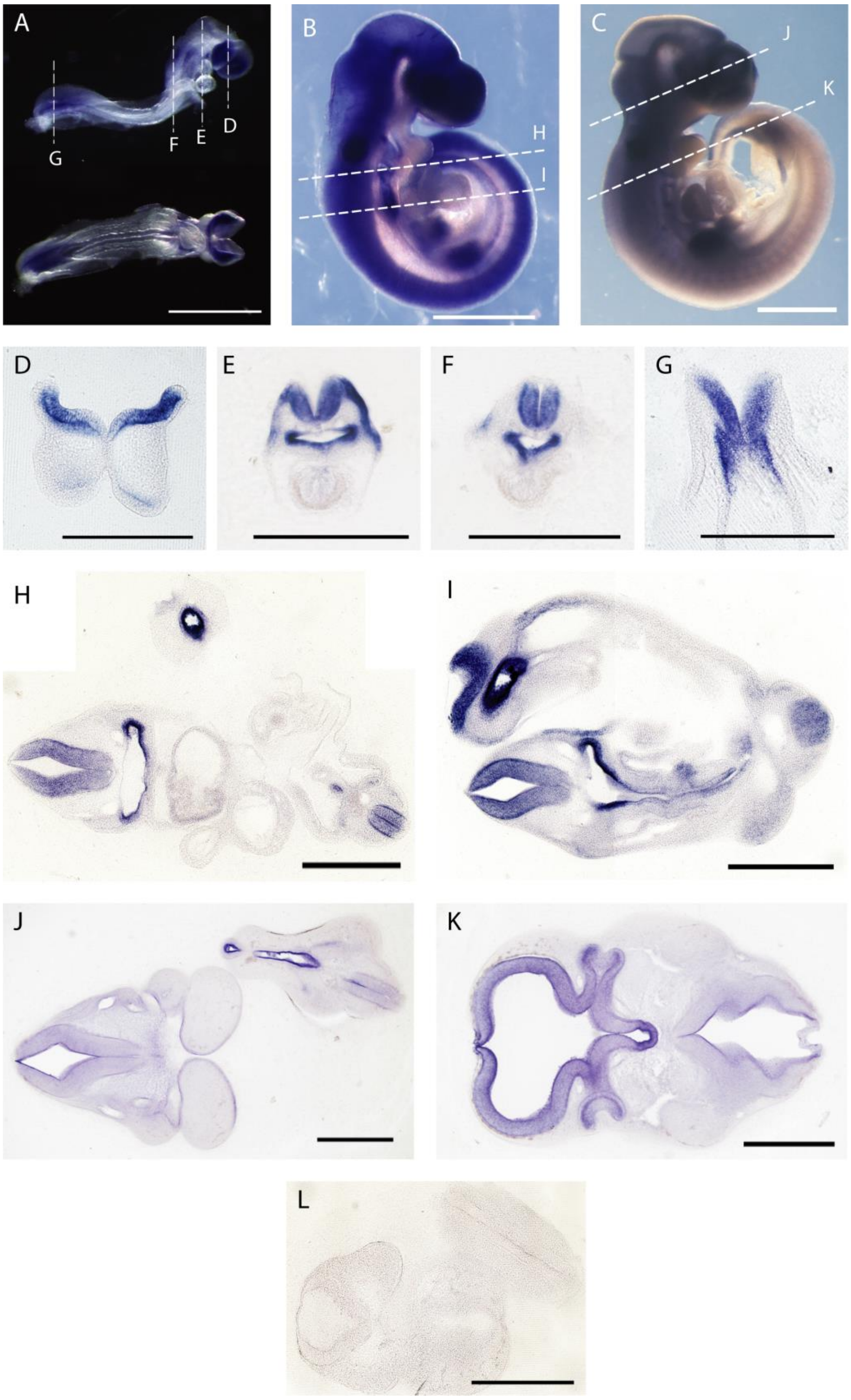

Supplementary Figure 1. Expression of *Gldc* at E8.5-10.5 in mouse

Gldc expression was detected by whole mount *in situ* hybridisation (A-C) and embryos were sectioned to reveal sites of expression (D-K). At E8.5 (A, D-G), E9.5 (B, H, I) and E10.5 (C, J, K) expression was abundant in the neural folds and closed neural tube with less intense expression around the foregut pocket (E, F) and limb buds (B, C, I). The sense probe (L) gave no specific signal. Dotted lines in A-C indicate axial level of sections in D-K. Scale bars represent 1 mm.

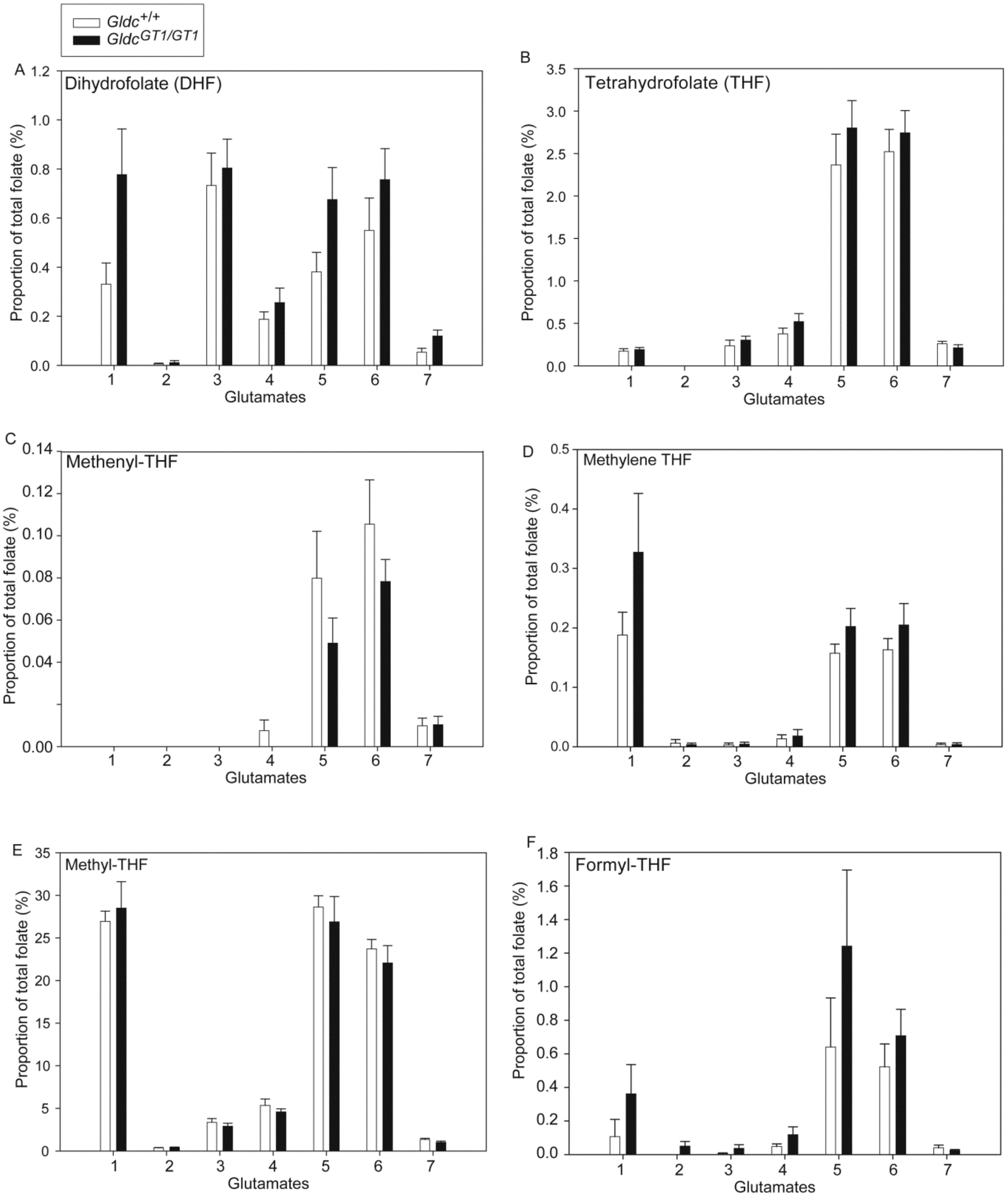

Supplementary Figure 2. Abundance of polyglutamated forms of folates in formate-treated *Gldc* mutant and wild-type embryos at E11.5. Significant differences between genotypes were not observed.

Metabolite	MRM	Cone voltage (V)	Collision Energy (V)
DHF GLU*	442.26 > 176.16	32.0	22.0
DHF GLU2	571.22 > 175.11	30.0	38.0
DHF GLU3	700.33 > 128.15	60.0	42.0
DHF GLU4	829.44 > 128.15	58.0	50.0
DHF GLU5	478.73 > 128.15	22.0	30.0
DHF GLU6	543.38 > 128.15	20.0	34.0
DHF GLU7	607.94 > 128.15	20.0	32.0
THF GLU*	444.26 > 315.19	60.0	22.0
THF GLU2	573.26 > 315.58	50.0	38.0
THF GLU3	702.37 > 128.15	60.0	42.0
THF GLU4	831.48 > 128.15	58.0	50.0
THF GLU5	479.75 > 128.15	22.0	30.0
THF GLU6	544.40 > 128.15	20.0	34.0
THF GLU7	608.96 > 128.15	20.0	32.0
CH-THF GLU*	454.22 > 281.21	60.0	30.0
CH- THF GLU2	583.18 > 281.36	50.0	38.0
CH- THF GLU3	712.29 > 128.15	65.0	42.0
CH- THF GLU4	841.40 > 128.15	58.0	50.0
CH-THF GLU5	484.71 > 128.15	22.0	30.0
CH- THF GLU6	549.36 > 128.15	20.0	34.0
CH-THF GLU7	613.92 > 128.15	20.0	32.0
CH2-THF GLU*	456.25 > 327.21	60.0	22.0
CH2-THF GLU2	585.35 > 127.90	50.0	38.0
CH2-THF GLU3	714.46 > 128.15	60.0	42.0
CH2-THF GLU4	843.57 > 128.15	58.0	50.0
CH2-THF GLU5	485.79 > 128.15	22.0	30.0
CH2-THF GLU6	550.45 > 128.15	20.0	34.0
CH2-THF GLU7	615.00 > 128.15	20.0	32.0
CH3-THF GLU*	458.26 > 329.22	54.0	22.0
CH3-THF GLU2	587.24 > 329.11	40.0	38.0
CH3-THF GLU3	716.35 > 128.15	60.0	42.0
CH3-THF GLU4	845.46 > 128.15	58.0	50.0
CH3-THF GLU5	486.74 > 128.15	22.0	30.0
CH3-THF GLU6	551.39 > 128.15	20.0	34.0
CH3-THF GLU7	615.90 > 128.15	20.0	32.0
CHO-THF GLU*	472.26 > 315.19	52.0	26.0
CHO-THFGLU2*	601.29 > 128.15	40.0	42.0
CHO-THFGLU3*	730.30 > 128.15	60.0	42.0
CHO-THFGLU4*	859.35 > 128.15	58.0	50.0
CHO-THFGLU5*	493.78 > 128.15	22.0	30.0
CHO-THFGLU6*	558.34 > 128.15	20.0	34.0
CHO-THF GLU7*	623.01 > 128.15	20.0	32.0

Supplementary Table 1. Parameters for multiple reaction monitoring for folate analysis. Folate standards (indicated with *) were analysed by multiple reaction monitoring (MRM) with optimised cone voltage and collision energy for precursor and product ions (Leung et al., *Mol. Cell Biochem.* **378**, 229-236, 2013). Where folate standards were not available, MRMs were calculated accordingly with reference to Garratt et al. (Rapid Commun Mass Spectrom 19: 2390-2398). DHF=Dihydrofolate; THF=Tetrahydrofolate; CH-THF= 5,10-Methenyl-THF; CH2-THF=5,10-Methylene-THF; CH3-THF=5-Methyl-THF;CHO-THF=10-Formyl-THF; and GLUn = number of glutamation.

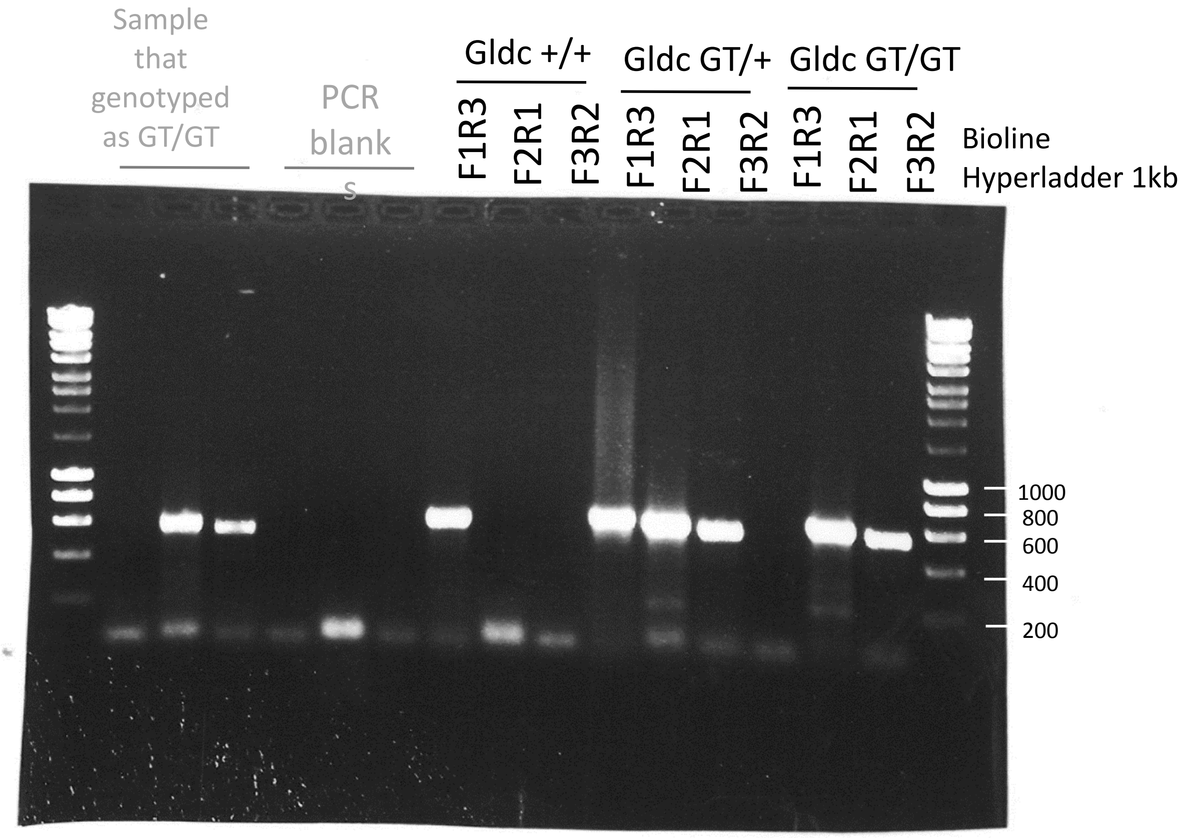

Full image of gel shown in Figure 1.
